# Non-contrast free-breathing 2D CINE compressed SENSE T1-TFE cardiovascular MRI at 3T in sedated young children for assessment of congenital heart disease

**DOI:** 10.1371/journal.pone.0297314

**Published:** 2024-02-08

**Authors:** Inka Ristow, Caroline-Viktoria Hancken-Pauschinger, Shuo Zhang, Maria Stark, Michael G. Kaul, Carsten Rickers, Jochen Herrmann, Gerhard Adam, Peter Bannas, Lennart Well, Julius Matthias Weinrich

**Affiliations:** 1 Department of Diagnostic and Interventional Radiology and Nuclear Medicine, University Medical Center Hamburg-Eppendorf, Hamburg, Germany; 2 Department of Diagnostic and Interventional Radiology and Nuclear Medicine, Section of Pediatric Radiology, University Medical Center Hamburg-Eppendorf, Hamburg, Germany; 3 Philips GmbH Market DACH, Hamburg, Germany; 4 Institute of Medical Biometry and Epidemiology, University Medical Center Hamburg-Eppendorf, Hamburg, Germany; 5 University Heart Center, Adult Congenital Heart Disease Unit, University Medical Center Hamburg-Eppendorf, Hamburg, Germany; University of Pisa, ITALY

## Abstract

Cardiac MRI is a crucial tool for assessing congenital heart disease (CHD). However, its application remains challenging in young children when performed at 3T. The aim of this retrospective single center study was to compare a non-contrast free-breathing 2D CINE T1-weighted TFE-sequence with compressed sensing (FB 2D CINE CS T1-TFE) with 3D imaging for diagnostic accuracy of CHD, image quality, and vessel diameter measurements in sedated young children. FB 2D CINE CS T1-TFE was compared with a 3D non-contrast whole-heart sequence (3D WH) and 3D contrast-enhanced MR angiography (3D CE-MRA) at 3T in 37 CHD patients (20♂, 1.5±1.4 years). Two radiologists independently assessed image quality, type of CHD, and diagnostic confidence. Diameters and measures of contrast and sharpness of the aorta and pulmonary vessels were determined. A non-parametric multi-factorial approach was used to estimate diagnostic accuracy for the diagnosis of CHD. Linear mixed models were calculated to compare contrast and vessel sharpness. Krippendorff’s alpha was determined to quantify vessel diameter agreement. FB 2D CINE CS T1-TFE was rated superior regarding image quality, diagnostic confidence, and diagnostic sensitivity for both intra- and extracardiac pathologies compared to 3D WH and 3D CE-MRA (all p<0.05). FB 2D CINE CS T1-TFE showed superior contrast and vessel sharpness (p<0.001) resulting in the highest proportion of measurable vessels (740/740; 100%), compared to 3D WH (530/620; 85.5%) and 3D CE-MRA (540/560; 96.4%). Regarding vessel diameter measurements, FB 2D CINE CS T1-TFE revealed the closest inter-reader agreement (Krippendorff’s alpha: 0.94–0.96; 3D WH: 0.78–0.94; 3D CE-MRA: 0.76–0.93). FB 2D CINE CS T1-TFE demonstrates robustness at 3T and delivers high-quality diagnostic results to assess CHD in sedated young children. Its ability to function without contrast injection and respiratory compensation enhances ease of use and could encourage widespread adoption in clinical practice.

## 1 Background

Congenital heart disease (CHD) is the most common congenital disorder with an incidence of about 8:1000 and a major contributor to infant morbidity and mortality [1] The outcome of children suffering from CHD has significantly improved [2]. Innovative developments in cardiac surgery and intervention but also continuous advances in imaging techniques have contributed to earlier and more accurate diagnosis of CHD [3].

Echocardiography is the first-line imaging method in children with CHD due to its non-invasiveness, wide availability, and cost-effectiveness [4, 5]. However, echocardiography is limited for the delineation of extracardiac structures, e.g., aortic and pulmonary vessels [6]. Computed tomography (CT) is often considered the modality of choice for preoperative imaging and permits fast and high-resolution scans but involves radiation exposure and the application of intravenous contrast [7–9].

Cardiovascular magnetic resonance (CMR) allows for standardized and reproducible measurements of thoracic vessels [10–14] as well as for structural and functional visualization of cardiac anatomy [3, 15] without ionizing radiation, though very often sedation is required in young children. MR angiography (MRA) provides high-quality morphological images and has demonstrated diagnostic utility in patients with CHD [2]. However, most MRA techniques require the administration of a gadolinium-based contrast agent [7, 16]. Contrast application is often omitted in young children due to controversies regarding potential retention in tissues [17].

Current non-contrast MRA techniques are typically based on balanced steady-state free precession (bSSFP) imaging, as also applied in standard CINE sequences for cardiac function analysis [3, 18–20]. However, MR-based assessment of the cardiothoracic vasculature in young children including infants poses additional difficulties due to small anatomical structures, complex flow conditions, and unstable respiratory status or the requirement for sedation [21]. The major drawback of cardiac bSSFP MRI in young children is high susceptibility due to local field inhomogeneities with the presence of flow or banding artifacts. Therefore, its application is limited, particularly at higher field strength ≥3T covering a large field of view [22]. Non-balanced MRI techniques have been proposed to circumvent this challenge [23, 24]. However, these non-bSSFP MRI techniques only provide static images and typically require synchronization of the image acquisition to patient respiration [23, 25] using either navigator echoes or MR-compatible cameras [26]. This results in complex operational procedures and prolonged scan time.

The aim of this study was to evaluate a non-contrast non-balanced free-breathing 2D CINE T1-weighted turbo-field echo (TFE) sequence with compressed sensing reconstruction (FB 2D CINE CS T1-TFE) at 3T in sedated young children with CHD in comparison to established 3D techniques with and without contrast enhancement for diagnostic accuracy, image quality, and quantitative vessel diameter measurements.

## 2 Materials and methods

### 2.1 Study population

This single-center retrospective paired diagnostic study was approved by the local ethics board (Ärztekammer Hamburg, Germany) with a waiver of informed consent (WF-033/21). All procedures complied with the local data protection guidelines as well as the Declaration of Helsinki.

We included 37 young children with suspected CHD (20 male, mean age 1.5±1.4 years, range: 0–4) examined by cardiac MRI from 2018–2020. Data were accessed for research purposes from 08/2021–11/2021. Inclusion criteria were < five years of age, sedation during MRI scan, availability of FB 2D CINE CS T1-TFE, and at least one of the reference sequences (3D WH and/or 3D CE-MRA). All patients had clinical indications for CMR. The mean heart rate of the cohort during the CMR was 102±21 bpm (range 72–135).

Of all included patients and according to the reference standard, 24 (64.9%) patients had intracardiac structural pathologies, 16 (43.2%) patients had pathologies of the great extracardiac vessels, and ten (27.0%) patients had pathologies of the small extracardiac vessels. Reference standard for the correct diagnosis of structural pathologies was derived from the patient chart including all diagnostic and interventional procedures relating to the CHD, i.e., echocardiograms, CT and MRI scans, and reports from cardiac catheterizations and operations. Individual CHD-related information about the performed diagnostic and therapeutic procedures as well as baseline characteristics (gender, age, weight, height, and main cardiac diagnoses) are provided in the supporting **S1 Table in S1 File**.

### 2.2 Imaging protocol

Imaging was performed on a clinical whole-body 3T MRI system (Ingenia, Philips Healthcare, Best, The Netherlands) with a 70 cm bore and using a standard 32-channel torso coil. For cardiothoracic vasculature assessment, the following pulse sequences were included: i) ECG-gated non-contrast free-breathing 2D CINE T1-weighted TFE imaging with compressed sensing reconstruction (FB 2D CINE CS T1-TFE), ii) ECG-gated 3D whole-heart sequence (3D WH) with dual-echo gradient-echo Dixon, and iii) 3D contrast-enhanced MR-angiography (3D CE-MRA).

FB 2D CINE CS T1-TFE was acquired in all patients (N = 37). For 22 patients (59.5%), both 3D WH and 3D CE-MRA served as a reference, whereas for nine patients (40.5%) only 3D WH and for six patients only 3D CE-MRA was acquired.

FB 2D CINE CS T1-TFE was applied with radiofrequency (RF) spoiling to destroy the residual transverse magnetization at the end of each repetition time, leading to a T1-weighted contrast [27, 28]. Multiple signal averages were performed to average out the bulk motion effect and to improve the signal-to-noise ratio (SNR). Compressed SENSE, employing compressed sensing together with coil sensitivity information, was used with a moderate acceleration factor to shorten the scan time [29]. Axial, para-sagittal, and coronal planes were acquired covering the entire heart and main cardiothoracic vessels. FB 2D CINE CS T1-TFE was obtained with a high temporal resolution and multiple cardiac phases using retrospective ECG-gating. Detailed imaging parameters are summarized in **Table 1**. FB 2D CINE CS T1-TFE and 3D WH were obtained without intravenous contrast administration. 3D CE-MRA was performed by manual infusion of gadoterate meglumine (Dotagraf® 0.5 mmol/ml, dose 0.3 ml/kg per manual intravenous administration).

**Table 1:** MR imaging pulse sequences.

	3D WH	3D CE-MRA	FB 2D CINE CS T1-TFE
Basic pulse sequence	T2prep dual-echo Dixon TFE	RF-spoiled FFE	RF-spoiled TFE
FOV [mm^2^]	265 × 300	300 × 200	250 × 200
Matrix ACQ	176 x 200	230 × 120	160 × 126
Voxel ACQ [mm^3^]	1.5 × 1.5 × 1.5	1.29 × 1.66 × 1.8	1.56 × 1.58 × 5
Voxel REC [mm^3^]	0.75 × 0.75 × 0.75	0.75 × 0.75 × 0.9	0.98 × 0.98 × 5
# of slices	~ 133	~ 100	16 to 20
FH spatial coverage [mm]*	100	80 to 100	80 to 100
TR / TE [ms]	4.4 / 1.43 and 2.6	4.2 / 1.86	3.8 / 2.4
Flip angle [deg]	10	35	10
Temporal resolution [ms] ^†^	120		33 to 34
Cardiac phases ACQ	-	-	15 to 18
Cardiac phases REC	-	-	22 to 26
ECG synchronization	End-diastolic triggering	n.a.	Retrospective gating
Respiratory compensation	Navigator	-	-
NSA	1	1	2
SENSE or C-SENSE	C-SENSE 4	SENSE 3.5 × 1.5	C-SENSE 3.4
Scan time [min]	~ 3	< 1	1 to 1.5 per orientation
Scan orientation	Axial	Coronal	Axial, para-sagittal, coronal
MPR	Coronal, para-sagittal	Axial, para-sagittal	-

* Adjusted individually according to patient size.

^**†**^ Corresponding to TFE duration.

TFE: turbo field echo, FFE: fast field echo, SENSE: sensitivity encoding, C-SENSE / CS: compressed SENSE, WH: whole heart, mDixon: modified Dixon, FOV: field of view, ACQ: acquired, REC: reconstructed, FH: feet head, TR: repetition time, TE: echo time, NSA: number of signal averages, MPR: multiplanar reformation.

### 2.3 Data analysis

#### 2.3.1 Diagnostic accuracy

Images were presented to the readers in random order and at different time points. Two attending radiologists (L.W. and J.M.W.) with eight and nine years of experience respectively in cardiovascular imaging independently diagnosed structural pathologies. Both readers were blinded to the reference standard, the clinicians’ information provided in the radiological request form as well as to the results of the other imaging sequences.

Since the diagnosis “congenital heart disease” can often include a combination of complex pathologies in various cardiovascular anatomical structures, diagnostic accuracy is not reported for each vessel individually but summarized for three pre-defined compartments, namely: i) intracardiac structures (e.g., atrial/ventricular septum, papillary muscles), ii) great extracardiac vessels (e.g., aorta, vena cava, main/ left/right pulmonary artery), and iii) small extracardiac vessels (e.g., pulmonary veins, (hemi)azygos vein).

Each reader first assessed the presence or absence of structural pathologies in each individual vessel. Next, each reader individually assigned present structural pathologies of each patient to one of the three anatomical compartments. Diagnosis within each of the three anatomical compartments was then compared with the reference standard to assess if readers detected structural pathologies in each of the three anatomical compartments completely, partially, or not at all. A true-positive result arose when the reader detected all present structural pathologies correctly. Otherwise, a false-negative result arose when the reader did not detect any present structural pathology. An inconclusive result arose when the reader partially detected present structural pathologies. According to the intention-to-diagnose principle [30], inconclusive results were considered false results. This led to a conservative estimation of diagnostic accuracy.

#### 2.3.2 Subjective image quality and diagnostic confidence ratings

Both readers independently assessed i) overall image quality and ii) quality of different vessels of interest (ascending aorta, supra-aortic vessels, pulmonary arteries, superior and inferior vena cava, pulmonary veins) in each of the three sequences (3D WH, 3D CE-MRA, FB 2D CINE CS T1-TFE). As performed by others [10, 11, 31], a multipoint Likert scale was applied for image quality rating: 1 = dataset was considered non-diagnostic, 2 = marked blurring of the structures, preventing a complete anatomical diagnosis, 3 = diagnostic quality, despite moderate blurring of cardiac and vascular structures, 4 = good diagnostic quality with mild blurring, 5 = excellent image quality allowing for sharp delineation of cardiovascular structures. According to Kits et al. (2021), diagnostic confidence for the detection or exclusion of pathologies was also rated on a five-point Likert scale: 1 = not at all confident, 2 = only slightly confident, 3 = neutral, 4 = predominantly confident, 5 = very confident [32].

#### 2.3.3 Quantitative image quality assessment

An in-house developed plugin (QMapIt) for ImageJ was used to semi-automatically assess MR image quality [33]. Quantitative vessel analysis was performed for all patients with complete datasets including all three sequences (N = 22) as already reported by others [10, 20]. First, two readers (I.R. and L.W.) independently placed a perpendicular line through the lumen of the ascending aorta and the main pulmonary artery. Subsequently, all signal intensities within this line were plotted on a parabolic graph. The maximum signal intensity and two minimum signal intensities on the left and right sides of the graph were determined automatically. To assess vessel sharpness, which corresponds to the transition of the vessel lumen to both vessel walls, the mean up- and down-slopes between two turning points of the parabolic-shaped signal intensity curves were automatically calculated. Contrast was defined as the absolute difference between the maximum signal intensity of the vessel lumen and the average of both minimum signal intensities of the left and right sides of the vessel wall.

#### 2.3.4 Vessel diameter measurements

Diameters of the aorta were independently measured on para-sagittal planes by both readers at seven predefined anatomic landmarks: 1) sinuses of Valsalva, 2) sinotubular junction, 3) ascending aorta (at the level of the main pulmonary artery), 4) transverse aortic arc (between brachiocephalic and left common carotid artery), 5) aortic isthmus, 6) distal arch, and 7) descending aorta (at the level of the main pulmonary artery). Diameters of the pulmonary artery were assessed on axial planes for the main, left and right pulmonary arteries. Readers were free to choose appropriate slices displaying the maximum profile of the respective vessel from the stacks. As for FB 2D CINE CS T1-TFE, readers were free to select the best slice at an appropriate systolic time interval which provided a correspondingly high signal in the vessel lumen.

#### 2.3.5 Statistical analysis

Sensitivity was chosen as the primary endpoint according to the guidelines on the evaluation of diagnostic tests by the European Medicines Agency and the U.S. Food and Drug Administration [34, 35]. Specificity was not considered as a co-primary endpoint because only true-negative test results were expected. Therefore, the comparison of specificities between the three sequences was not meaningful.

There are three primary hypotheses structured in hierarchical order, which state that sensitivity for the diagnosis of structural pathologies of FB 2D CINE CS T1-TFE is superior to the sensitivity of 3D CE-MRA, with respect to the three anatomical compartments: i) intracardiac structures, ii) great extracardiac vessels, and iii) small extracardiac vessels.

Secondary hypotheses state that the sensitivity for the diagnosis of structural pathologies of FB 2D CINE CS T1-TFE is superior to the sensitivity of 3D WH with respect to the three compartments. Additionally, predictive values of each sequence within each structure, differences in image quality, diagnostic confidence between the sequences, and agreement of diameters between both readers within each sequence were considered.

Statistical analysis was performed using the software R, version 4.0.5 (R Foundation for Statistical Computing, Vienna, Austria) [36]. The significance level was set to 5% two-sided. To adjust for multiple testing, the three primary hypotheses were structured in hierarchical order. Due to this structure, subordinate primary hypotheses may only be evaluated, if all superordinate primary hypotheses lead to a significant test result. Otherwise, the evaluation of subordinate hypotheses is performed descriptively, just like the analysis of secondary hypotheses.

To analyze diagnostic accuracy in this partially crossed study design, a non-parametric multi-factorial approach [37] was used to report diagnostic accuracy measures with two-sided 95% Wald confidence intervals (CI) for each sequence and each anatomical compartment averaged over both readers. To analyze primary and key secondary hypotheses referring to the comparison of sensitivities between sequences within one anatomical compartment, differences with two-sided 95% Wald CI between sensitivities of FB 2D CINE CS T1-TFE and 3D WH or 3D CE-MRA were calculated, respectively. A significant test result was obtained when the CI of the according difference between both sensitivities did not cover a difference of 0% and the sensitivity of FB 2D CINE CS T1-TFE was higher than the one of the comparator sequences. In this case, superiority of the sensitivity of FB 2D CINE CS T1-TFE compared to one of the comparator sequences was concluded regarding our primary hypotheses. Boxplots are shown to compare image quality and diagnostic confidence ratings between sequences, and overall comparisons between all sequences were additionally performed using non-parametric Friedman tests. Furthermore, pairwise comparisons with the non-parametric Wilcoxon test between FB 2D CINE CS T1-TFE and 3D WH or 3D CE-MRA are provided, respectively. To quantify the agreement between the two readers in measuring vessel diameters in the same sequence, the differences between measurements of both readers of the same vessel were calculated and depicted as boxplots. Additionally, Krippendorff’s alpha with bootstrapped 95%-CI was calculated to determine the reliability of the two readers of the same vessel assessed with the same sequence [38]. To compare quantitative image quality between the three sequences, two linear mixed models were calculated with either contrast or slope as the dependent variable (R-package *lmerTest* version 3.1–3) [39]. Fixed effects were MRI sequence, vessel, and reader. Regarding the model with slope as dependent variable, side of the slope was additionally included as a fixed effect. A random intercept for each patient was modeled. Marginal means and corresponding contrasts with two-sided 95%-CI for pairwise comparison of the sequences were estimated (R-package *emmeans* version 1.7.2) [40].

## 3 Results

### 3.1 Diagnostic accuracy

The proportion of correct diagnoses was highest in FB 2D CINE CS T1-TFE across both readers: Regarding intracardiac structures, 44 out of 48 pathologies were correctly diagnosed with FB 2D CINE CS T1-TFE (91.7%), and four diagnoses were partially correct (8.3%). With 3D WH correct diagnoses were made in 19 pathologies (52.8%), four diagnoses were only partially correct (11.1%), and 13 were false-negative (36.1%). With 3D CE-MRA correct diagnoses were made in 13 pathologies (36.1%), four diagnoses (11.1%) were only partially correct, and 19 were false-negative (52.8%), (supporting S2 **Table in S1 File**).

Regarding the great extracardiac vessels, 32 out of 32 pathologies were correctly diagnosed with FB 2D CINE CS T1-TFE (100%). With 3D WH correct diagnoses were made in 15 cases (62.5%), two diagnoses were only partially correct (8.33%), and seven were false-negative (29.2%). With 3D CE-MRA correct diagnoses were made in 16 cases (57.1%), two diagnoses (7.1%) were only partially correct, and ten were false-negative (35.7%), (supporting **S3 Table in S1 File**).

Regarding the small extracardiac vessels, 18 out of 20 pathologies were correctly diagnosed with FB 2D CINE CS T1-TFE (90.0%), one diagnosis was only partially correct (5.0%), and one diagnosis was considered wrong (5.0%). With 3D WH correct diagnoses were made in three pathologies (18.8%), one diagnosis was only partially correct (6.3%), and twelve diagnoses were false-negative (75.0%). With 3D CE-MRA correct diagnoses were made in six pathologies (42.9%), eight pathologies were false-negative (57.1%), (supporting **S4 Table in S1 File**).

**Fig 1:**
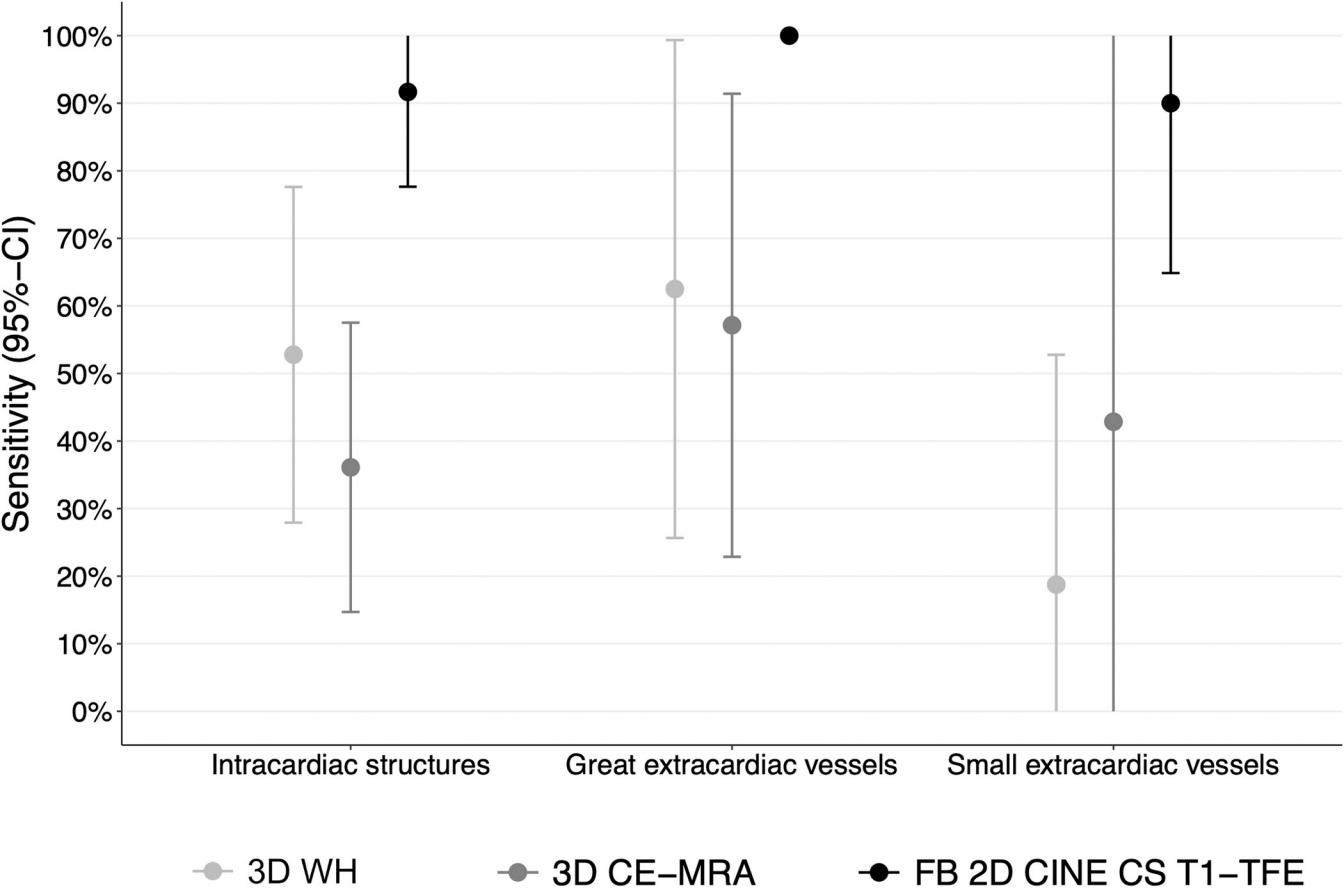
Comparison of mean sensitivities for detection of CHD. FB 2D CINE CS T1-TFE shows significant superiority of sensitivity in intracardiac structures and great extracardiac vessels compared to the sensitivity of 3D CE-MRA because 95% confidence intervals of the difference in sensitivities between both sequences did not cover a difference of 0%. In small extracardiac vessels, no statistically significant differences between the sensitivities of FB 2D CINE CS T1-TFE and 3D CE-MRA were identified because the 95% confidence interval of the difference in sensitivities between both sequences is wide and therefore includes a difference of 0%. If confidence intervals are not range-preserving, they are cut at 0% or 100% and depicted without lower or upper boundaries. CI = confidence interval.

Sensitivities and negative predictive values for the three anatomical compartments averaged over both readers are reported in **Table 2**. FB 2D CINE CS T1-TFE showed significant superiority of sensitivity in intracardiac structures compared to the sensitivity of 3D CE-MRA (**Fig 1**). Mean sensitivity for the detection of intracardiac pathologies was 91.7% in FB 2D CINE CS T1-TFE (95%-CI: 77.6–100%), 52.8% in 3D WH (95%-CI: 27.9–77.6%), and 36.1% in 3D CE-MRA (95%-CI: 14.7–57.5%). Likewise, FB 2D CINE CS T1-TFE showed significant superiority regarding great extracardiac vessels compared to the sensitivity of 3D CE-MRA. Mean sensitivity for the detection of pathologies of the great extracardiac vessels was 100% in FB 2D CINE CS T1-TFE, 62.5% in 3D WH (95%-CI: 25.7–99.3%), and 57.1% in 3D CE-MRA (95%-CI: 22.9–91.4%).

**Table 2:** Sensitivity and negative predictive values for diagnosis of intracardiac and extracardiac pathologies.

Anatomical compartment	3D WH-mDixon		3D CE-MRA		FB 2D CINE CS T1-TFE	
Intracardiac structures	Sensitivity	52.8% (27.9–77.6%)	Sensitivity	36.1% (14.7–57.5%)	Sensitivity	91.7% (77.6–100%)
	NPV	61.3% (44.6–78.7%)	NPV	46.7% (34.7–58.8%)	NPV	86.7% (58.7–100%)
Great extracardiac vessels	Sensitivity	62.5% (25.7–99.3%)	Sensitivity	57.1% (22.9–91.4%)	Sensitivity	100%
	NPV	80.9%	NPV	70.2%	NPV	100%
Small extracardiac vessels	Sensitivity	18.8% (0–52.8%)	Sensitivity	42.9% (0–100%)	Sensitivity	90% (64.9–100%)
	NPV	78.0%	NPV	84.1%	NPV	96.6%

Displayed are mean sensitivities and negative predictive values (NPV) with 95% confidence intervals. Results were averaged across both readers. There were no false positive diagnoses. Consequently, both specificity and positive predictive value (PPV) were 100% in each sequence.

In small extracardiac vessels, no statistically significant differences between the sensitivities of FB 2D CINE CS T1-TFE and 3D CE-MRA were identified. However, the estimated sensitivity of FB 2D CINE CS T1-TFE (90.0%, 95%-CI: 64.9–100.0%) was higher compared to the estimated sensitivity of 3D CE-MRA (42.9%, 95%-CI: 0–100%). The mean sensitivity of 3D WH was 18.8% (95%-CI: 0–52.8%). Regarding secondary comparisons between FB 2D CINE CS T1-TFE and 3D WH, the estimated sensitivities of FB 2D CINE CS T1-TFE are higher than those of 3D WH (**Table 2**, **Fig 1**).

Regarding intracardiac structures, differences between sensitivities were 55.6% (95%-CI: 32.4–78.7%) for FB 2D CINE CS T1-TFE - 3D CE-MRA and 38.9% (95%-CI:16.5–61.2%) for FB 2D CINE CS T1-TFE - 3D WH. Regarding great extracardiac vessels, differences between sensitivities were 42.9% (95%-CI: 8.6–77.1%) for FB 2D CINE CS T1-TFE - 3D CE-MRA and 37.5% (95%-CI: 0.7–74.3%) for FB 2D CINE CS T1-TFE - 3D WH, whereas differences regarding small extracardiac vessels were 47.1% (95%-CI: 0–100%) for FB 2D CINE CS T1-TFE - 3D CE-MRA and 71.3% (95%-CI: 34.0–100%) for FB 2D CINE CS T1-TFE - 3D WH.

### 3.2 Subjective image quality and diagnostic confidence ratings

Time-resolved FB 2D CINE CS T1-TFE cardiovascular MRI of an exemplary patient (male, 3 years old) with a superior sinus venosus atrial septal defect is depicted in the supporting movie **(S3 File).** Exemplary images for each of the multipoint Likert scale image quality degree are provided in supporting **S5 Fig in S1 File**.

**Fig 2:**
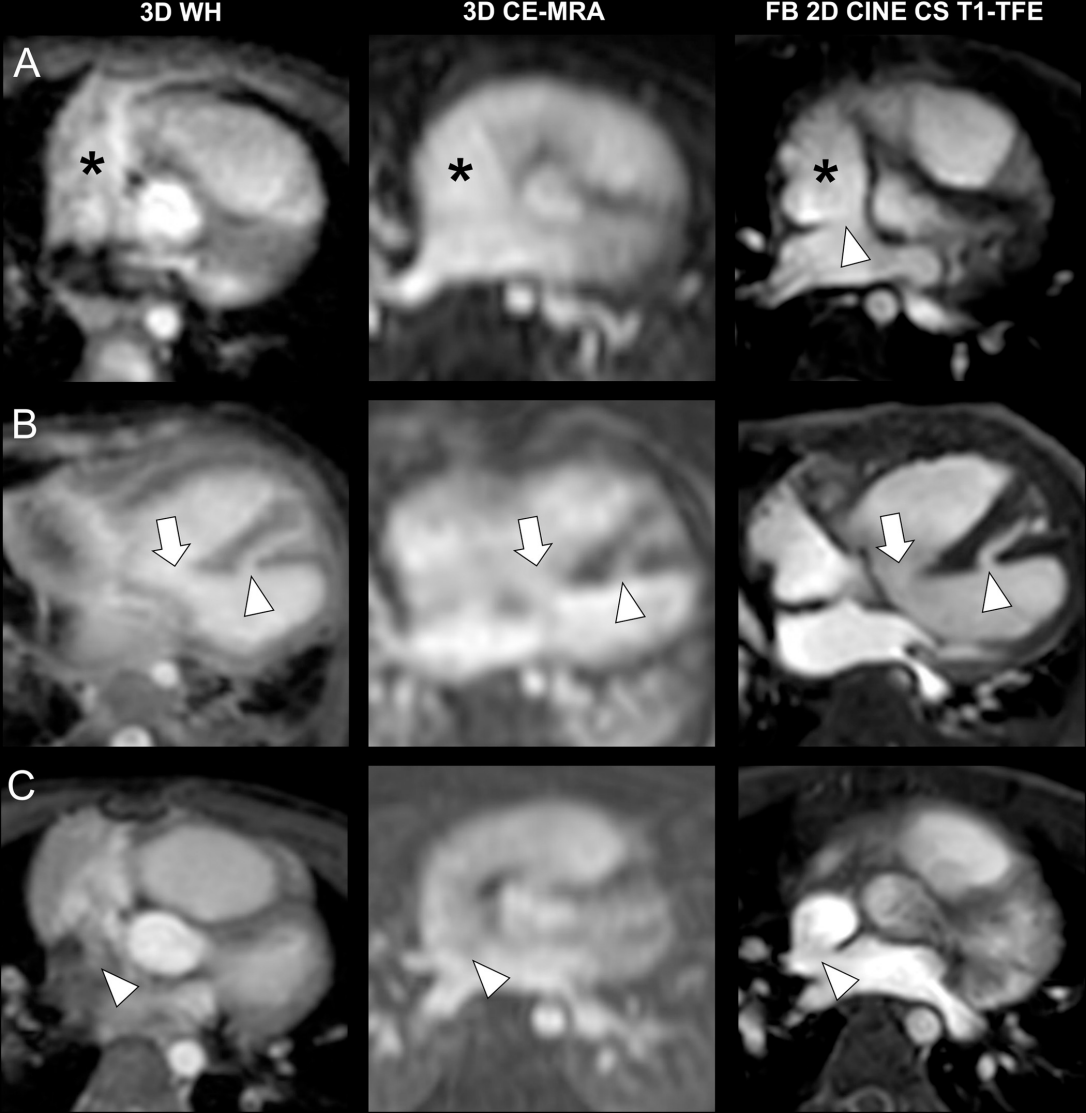
Intraindividual comparison regarding intracardiac structures in three different patients. **A)** Transverse slices in a three-year-old girl with an atrial septal defect (arrowhead). Asterisk indicates the right atrium. **B)** Transverse slices in a three-month-old boy with two ventricular septal defects (arrow + arrowhead). **C)** Transverse slices in a three-year-old boy with a superior sinus venosus atrial septal defect (arrowhead).

**Fig 3:**
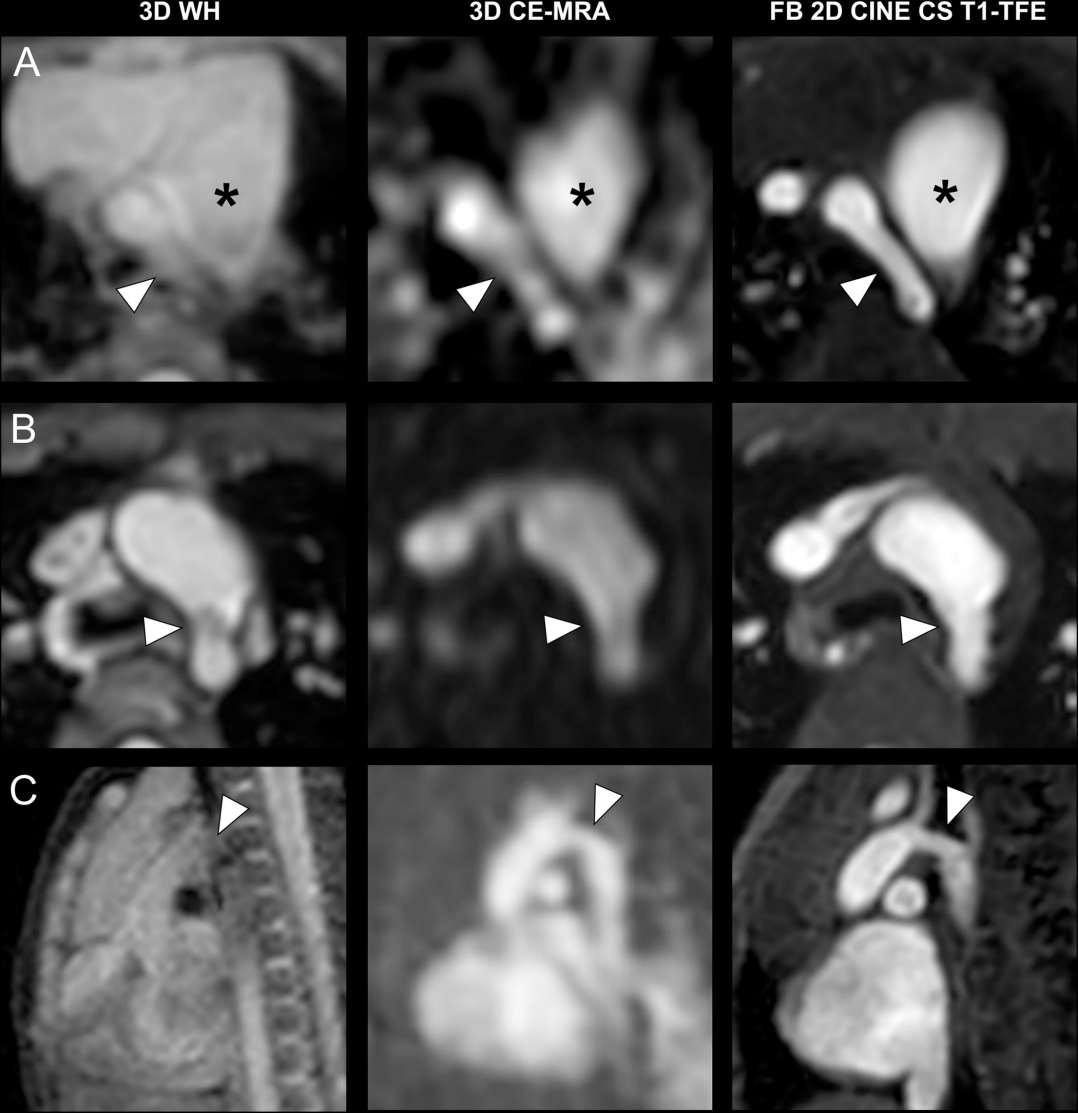
Intraindividual comparison regarding the great extracardiac vessels in three different patients. **A)** Transverse slices in a three-month-old boy with a hypoplastic aortic arch (arrowhead). Asterisk indicates the main pulmonary artery. **B)** Transverse slices in a three-year-old boy with a coarctation of the aorta (arrowhead). **C)** Para-sagittal slices in a three-month-old boy with a hypoplastic aortic arch (arrowhead).

**Fig 4:**
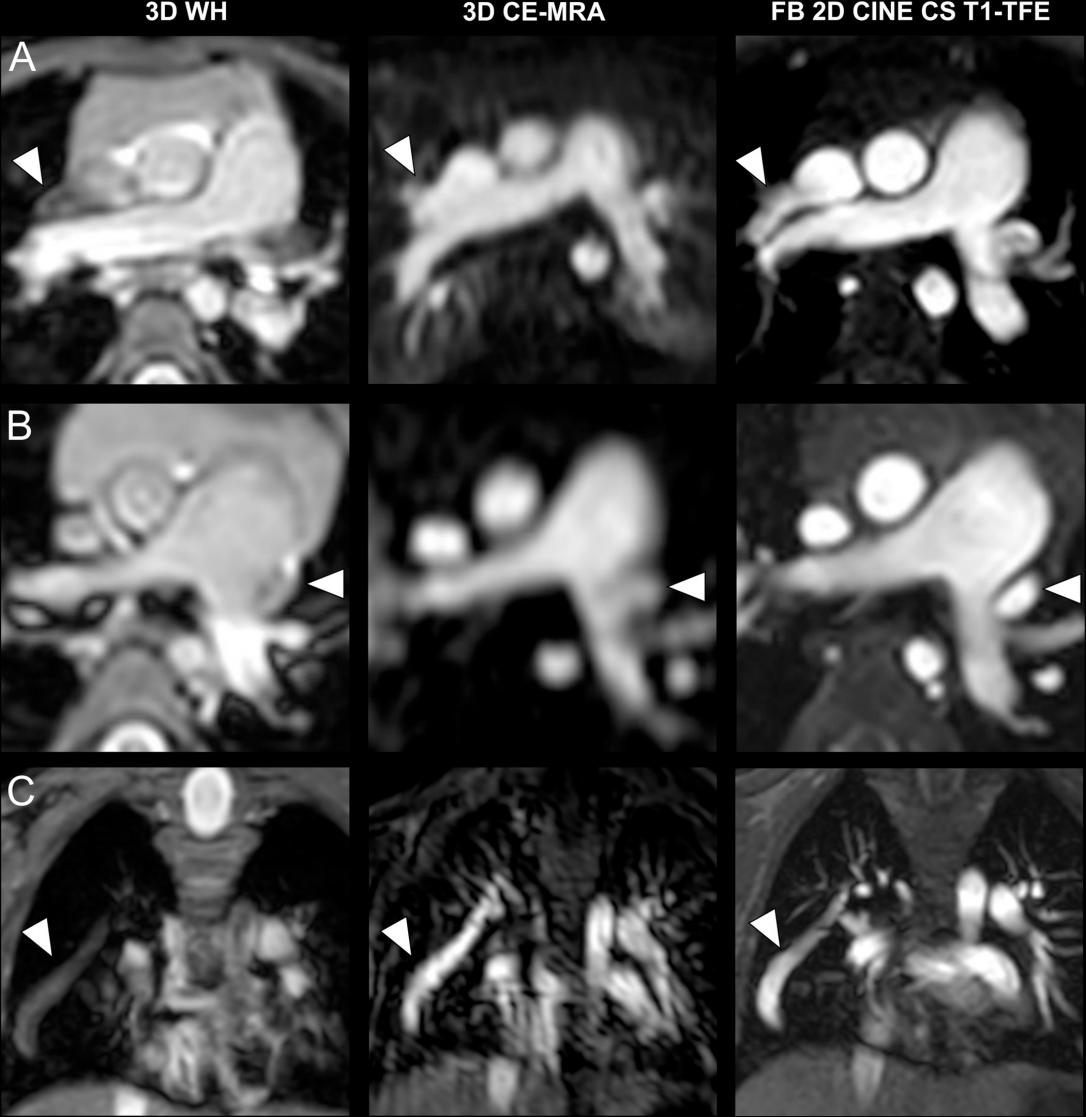
Intraindividual comparison regarding small extracardiac vessels in three different patients. **A)** Transverse slices in a three-year-old boy with a partial anomalous pulmonary venous connection of the right upper pulmonary vein to the superior vena cava (arrowhead). **B)** Transverse slices in a three-year-old girl with a persistent left superior vena cava (arrowhead). **C)** Coronal slices in a three-year-old girl with Scimitar syndrome. Arrowhead indicates anomalous venous return from the right lung to the inferior vena cava.

Representative images of patients with intracardiac pathologies are displayed in **Fig 2**. FB 2D CINE CS T1-TFE demonstrated superior image quality for the delineation of intracardiac structures compared to both 3D WH and 3D CE-MRA. In the same way, FB 2D CINE CS T1-TFE yielded the sharpest delineation of both great extracardiac (**Fig 3**) as well as small extracardiac vasculature (**Fig 4**).

**Fig 5:**
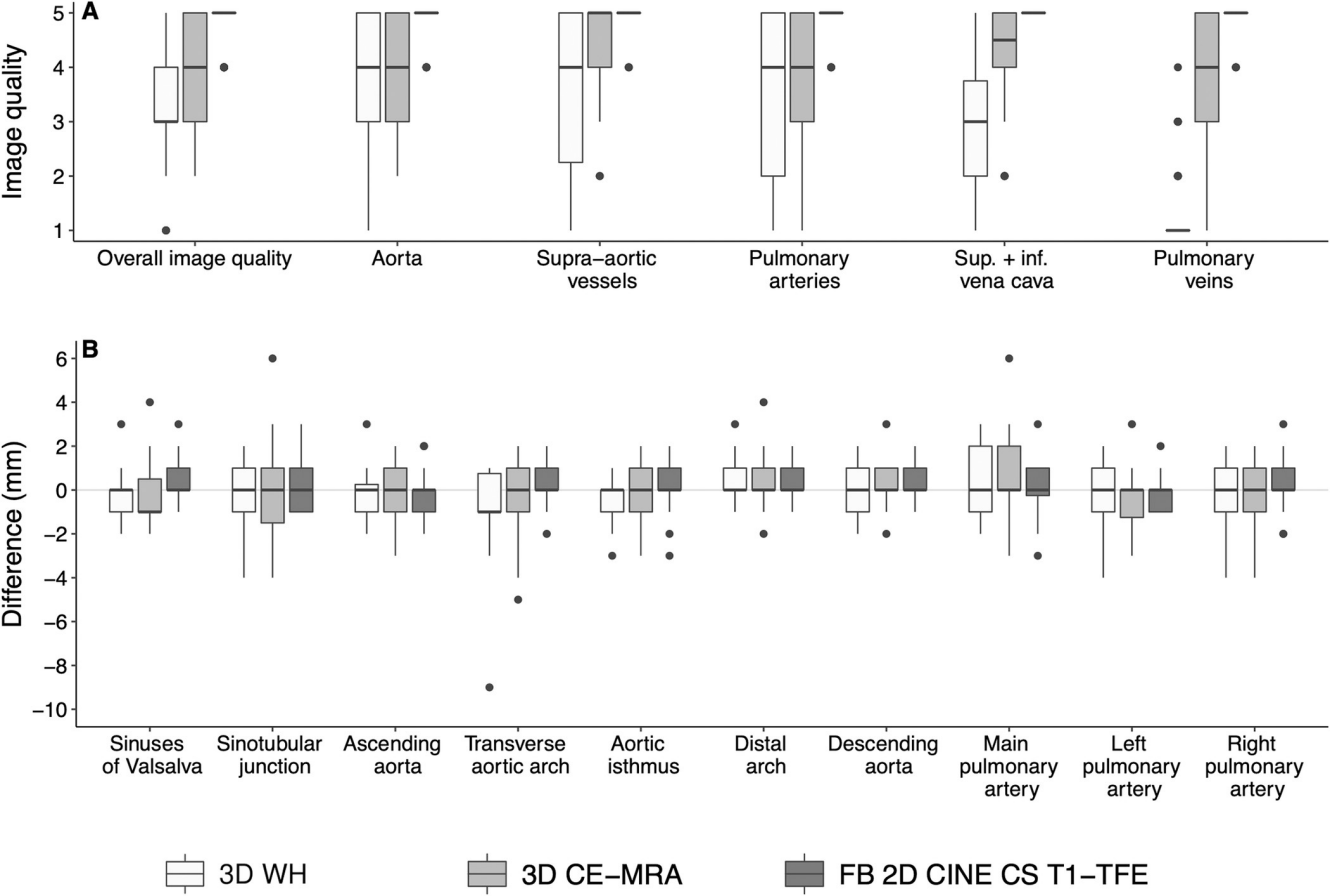
Image quality ratings and vessel diameter measurement agreements. **A)** Boxplots display image quality scores on a 5-point Likert scale for 3D WH, 3D CE-MRA, and FB 2D CINE CS T1-TFE. FB 2D CINE CS T1-TFE was rated superior for overall image quality (all p < .001) and image quality of all vascular structures of interest. **B)** Boxplots display differences in diameter measurements between the two readers at ten defined landmarks of the aorta and pulmonary vessels. Retrospective workup of the -9 mm outlier at the level of the transverse aortic for 3D WH revealed that the patient had aortic isthmus stenosis.

Medians with 25% and 75% quantiles of image quality ratings are displayed in **Table 3** and visualized in **Fig 5A**. Friedmann tests for the overall comparison of quality ratings between the three sequences and both readers yielded p-values <0.001. FB 2D CINE CS T1-TFE image quality was rated significantly higher compared to 3D WH as well as to 3D CE-MRA with respect to all vessels of interest (ascending aorta, supra-aortic vessels, pulmonary arteries, superior and inferior vena cava, and pulmonary veins) by both readers (all p<0.05; **Table 3**). Diagnostic confidence ratings were higher for FB 2D CINE CS T1-TFE compared to 3D WH (both readers p<0.001) and 3D CE-MRA (reader 1: p<0.001; reader 2: p≤0.016).

**Table 3:** Image quality and diagnostic confidence ratings.

Parameter	Value Likert Scale Median (25%, 75% Quantile)			Friedman-Test	Post-hoc Wilcoxon-Test	
	*3D WH mDixon (N = 31)*	*3D CE-MRA (N = 28)*	*FB 2D CINE CS T1-TFE (N = 37)*		*3D WH-mDixon vs*. *FB 2D CINE CS T1-TFE*	*3D CE-MRA vs*. *FB 2D CINE CS T1-TFE*
Overall	R1: 3.0 (3.0, 4.0)	R1: 4.0 (3.0, 5.0)	R1: 5.0 (5.0, 5.0)	χ^2^ = 76.1; N = 22;	R1: p< 0.001	R1: p< 0.001
	R2: 3.0 (2.5, 3.0)	R2: 4.0 (3.0, 5.0)	R2: 5.0 (5.0, 5.0)	df = 5; p< 0.001	R2: p< 0.001	R2: p< 0.001
Aorta	R1: 4.0 (3.0, 5.0)	R1: 4.0 (3.0, 5.0)	R1: 5.0 (5.0, 5.0)	χ^2^ = 34.6; N = 22;	R1: p< 0.001	R1: p = 0.001
	R2: 4.0 (2.5, 5.0)	R2: 4.0 (3.0, 5.0)	R2: 5.0 (5.0, 5.0)	df = 5; p< 0.001	R2: p< 0.001	R2: p< 0.001
Supra-aortic vessels	R1: 4.0 (3.0, 5.0)	R1: 5.0 (3.8, 5.0)	R1: 5.0 (5.0, 5.0)	χ^2^ = 24.7; N = 22;	R1: p< 0.001	R1: p = 0.009
	R2: 5.0 (2.0, 5.0)	R2: 5.0 (5.0, 5.0)	R2: 5.0 (5.0, 5.0)	df = 5; p< 0.001	R2: p< 0.001	R2: p = 0.034
Pulmonary arteries	R1: 4.0 (2.2, 5.0)	R1: 4.0 (3.0, 5.0)	R1: 5.0 (5.0, 5.0)	χ^2^ = 33.4; N = 22;	R1: p< 0.001	R1: p< 0.001
	R2: 5.0 (2.5, 5.0)	R2: 4.5 (3.0, 5.0)	R2: 5.0 (5.0, 5.0)	df = 5; p< 0.001	R2: p< 0.001	R2: p< 0.001
Vena cava (sup. + inf.)	R1: 3.0 (1.5, 3.5)	R1: 4.0 (3.0, 5.0)	R1: 5.0 (5.0, 5.0)	χ^2^ = 76.7; N = 22;	R1: p< 0.001	R1: p< 0.001
	R2: 3.0 (2.0, 3.5)	R2: 5.0 (4.0, 5.0)	R2: 5.0 (5.0, 5.0)	df = 5; p< 0.001	R2: p< 0.001	R2: p = 0.008
Pulmonary veins	R1: 1.0 (1.0, 1.0)	R1: 4.0 (2.0, 4.0)	R1: 5.0 (5.0, 5.0)	χ ^2^ = 91.9; N = 21;	R1: p< 0.001	R1: p< 0.001
	R2: 1.0 (1.0, 1.0)	R2: 4.0 (3.0, 5.0)	R2: 5.0 (5.0, 5.0)	df = 5; p< 0.001	R2: p< 0.001	R2: p< 0.001
Diagnostic confidence	R1: 3.0 (3.0, 4.0)	R1: 3.0 (3.0, 4.0)	R1: 5.0 (4.0, 5.0)	χ^2^ = 47.6; N = 21;	R1: p< 0.001	R1: p< 0.001
	R2: 4.0 (3.0, 5.0)	R2: 4.5 (4.0, 5.0)	R2: 5.0 (5.0, 5.0)	df = 5; p< 0.001	R2: p< 0.001	R2: p = 0.016

R1: reader 1; R2: reader 2; image quality rating 1 = dataset was considered non-diagnostic, 5 = excellent image quality allowing for sharp delineation of cardiovascular structures; diagnostic confidence rating 1 = not confident, 5 = completely confident).

### 3.3 Quantitative image quality assessment

Estimates of regression coefficients with 95%-CI and p-values of mixed models to compare contrast and slopes are provided in the supporting **S6 Table in S1 File**. Exemplary parabolic graph plots of the signal intensities in the ascending aorta of a 3-year-old boy are displayed in the supporting **S7 Fig in S1 File**. Estimated marginal means (averaged over both readers and vessels) for the contrast of the edges of the vessel lumen were 0.29 (95%-CI: 0.24–0.33) for 3D WH, 0.42 (95%-CI: 0.38–0.47) for 3D CE-MRA, and 0.65 (95%-CI: 0.60–0.69) for FB 2D CINE CS T1-TFE. Regarding vessel sharpness, estimated marginal means (averaged over both readers, vessels, and sides of up-and down-slopes of the signal intensity curves) were 279.51 (223.38–335.64) for 3D WH, 386.84 (330.91–442.76) for 3D CE-MRA, and 781.09 (725.17–837.02) for FB 2D CINE CS T1-TFE. Pairwise comparisons revealed higher contrast as well as steeper slopes of the signal intensity curves of the FB 2D CINE CS T1-TFE compared with 3D WH and 3D CE-MRA (all p<0.0001) (supporting **S8A, S8B Fig in S1 File**).

### 3.4 Vessel diameter measurements

All vessels (740/740) could be detected and measured with the FB 2D CINE CS T1-TFE, whereas 96.4% (540/560) of vessels could be detected and measured with the 3D CE-MRA and only 85.5% (530/620) with the 3D WH due to poor image quality.

Vessel diameter measurements assessed in the FB 2D CINE CS T1-TFE achieved higher reliability between both readers compared with the other two sequences (range of Krippendorff’s alpha (95%-CI of minimum/maximum) FB 2D CINE CS T1-TFE: 0.94 (0.86–0.97)– 0.96 (0.91–0.98); 3D WH 0.78 (0.46–0.89)– 0.94 (0.86–0.98); 3D CE-MRA: 0.76 (0.35–0.92)– 0.93 (0.88–0.96). Only for the ascending aorta approximately equivalently reliable measurements were observed in 3D WH and FB 2D CINE CS T1-TFE (supporting **S9 Table in S1 File**). Differences in diameter measurements between the two readers are displayed in **Fig 5B**.

## 4 Discussion

In this study, we evaluated a 2D non-contrast free-breathing T1-weighted turbo-field echo sequence with compressed sensing reconstruction for assessment of the cardiothoracic vasculature in sedated young children with congenital heart disease. Compared to established 3D techniques, the proposed FB 2D CINE CS T1-TFE method yielded significantly higher image quality allowing for superior delineation of both intracardiac and extracardiac structures with higher diagnostic confidence ratings, resulting in higher sensitivity for detection of CHD.

The superior image quality of vascular structures for FB 2D CINE CS T1-TFE mostly owes to a joint effect of its high temporal resolution and blood-to-tissue contrast, in comparison to 3D WH and 3D CE-MRA. Whereas all three techniques had similar in-plane spatial resolutions, ECG-gated FB 2D CINE CS T1-TFE with a temporal resolution of <33 ms was able to mitigate temporal blurring caused by cardiac motions, compared to 3D WH with a lower temporal resolution of 120 ms and 3D CE-MRA without ECG-gating. Both 3D techniques were susceptible to motion-induced data inconsistencies, such as from respiration, due to segmented *k*-space data filling over multiple cardiac and breathing cycles. In contrast, multiple signal averaging of periodic and steady respiratory cycles in sedated young children effectively compensated motion in FB 2D CINE CS T1-TFE, as shown in the early work in pediatric patients with CHD [41]. In addition, an approximately one-minute single scan time per orientation in T1-TFE accelerated by C-SENSE also helped to mitigate motion, as compared to a three-minute volumetric scan in 3D WH. On the other hand, 3D techniques with volume- or non-selective excitation hamper tissue differentiation, especially when no contrast agent is applied. For FB 2D CINE CS T1-TFE, high contrast can be obtained due to the generic 2D inflow effect, as shown in **Figs 2–4**. All these combined effects have led to higher diagnostic confidence ratings and diagnostic sensitivity in 2D FB T1-TFE for both intra- and extracardiac pathologies, compared to 3D WH and 3D CE-MRA.

The applied FB 2D CINE CS T1-TFE MRI sequence employed RF spoiling with a T1-weighted contrast [41, 42]. Fully balanced gradient-echo sequences with high flip angles in a range of 45˚ to 80˚, as typically used in conventional cardiac CINE MRI in adults, are highly sensitive to resonance offset effects. In contrast, RF-spoiled gradient-echo exploits a generic T1-weighted contrast and inflow effect with lower flip angles in a range of only 5˚ to 20˚, which is less critical to RF power deposit as well as local field inhomogeneities at 3T [27, 28]. This may be further exploited to assess prosthetic materials such as metal stents or clips in terms of artifacts, although none of the patients in this study met these criteria. Similarly, the generic T1 contrast may exhibit an additional advantage after contrast injection, which needs to be studied separately.

So far different variants of Dixon-based techniques have been proposed for non-contrast-enhanced non-balanced 3D WH morphological imaging at 3T [23, 24, 43]. These techniques have shown promise for the depiction of intra- and extracardiac structures. However, their drawbacks typically include limited blood-to-tissue contrast for certain anatomical structures and possible water-fat-swap artifacts [43, 44]. These are also demonstrated in the present study, e.g., in the right atrium (**Fig 2C**), the aortic arch (**Fig 3C**), and the pulmonary veins (**Fig 4A**). In fact, similar techniques have been reported with contrast administration for improved tissue contrast [44, 45]. In addition, Dixon-based techniques only provide static images at typically end-diastolic phase. In the current work, we did not perform volumetric and cardiac functional analysis with the FB 2D CINE CS T1-TFE due to too few cases with standard cine bSSFP for comparison, although a high temporal resolution with multiple cardiac phases was obtained. This needs to be investigated in future studies. The sensitivity of the two 3D sequences seemed generally low compared with the current clinical performance of CMR. This may be due to several reasons including limited clinical information provided to the radiologists across different study designs or limited comparability among the sequence variants. For example, many centers tend to use non-3D bSSFP and/or IR-GRE techniques for the diagnosis of CHD.

Nevertheless, current results may suggest a complete cardiovascular workup for young children with CHD, even in very young, sedated children, based on native free-breathing T1-TFE in two or three imaging planes in the clinical practice. In addition to the diagnosis of structural pathologies, the obtained cine movies may provide further information about hemodynamics, such as valve insufficiencies or stenoses, with even potential analysis for cardiac volumes and functions. In particular, with regard to the diagnosis of malformations of the small thoracic vessels such as the pulmonary veins, FB 2D CINE CS T1-TFE is clearly superior to the native 3D WH comparator sequence. For example, the two readers detected only 18.8% of small thoracic vessel pathologies in the 3D WH sequence, whereas 90% of the structural pathologies were detected in the FB 2D CINE CS T1-TFE. Recent technical advances have allowed 3D cine in free breathing with [46] or without contrast administration [47] in adults. Although not yet clinically available, they may offer promising utility in assessing cardiothoracic vasculature in this patient cohort because of their ability to obtain multiplanar reformations.

In addition to the presented FB 2D CINE CS T1-TFE, the use of 2D black blood imaging can be considered a complementary imaging technique for the assessment of cardiac anatomy, which has proven to be particularly helpful in the diagnosis of vascular structures at high spatial resolution with low magnetic susceptibility artifacts [48–51].

This study had the following limitations: First, the blinded study design used in this study reflects clinical routine only to a limited extent. In contrast to the unspecific term “diagnosis of CHD”, far more specific clinical questions are usually posed to the radiologist, which are to be answered by means of CMR. Furthermore, these clinical information and questions significantly influence the choice of the measurement protocol.

Second, for some of the patients included, only two of the three sequences were acquired. This limitation is due to the retrospective design of the study: in our clinical routine, an attending radiologist partakes in the scan process and decides to waive the need for a CE-MRA in patients with already clearly depicted pathology in the non-contrast sequences. Moreover, it would also be desirable to compare the FB 2D CINE CS T1-TFE with a standard CINE SSFP.

Third, it should be considered that the use of the intention-to-diagnosis approach as a rather conservative approach to face inconclusive test results, may somewhat underestimate reported sensitivities since partially correct diagnoses within an anatomical compartment were treated as a misdiagnosis in statistical analyses.

Fourth, the reference standard was defined as the final diagnosis, which was derived from all available diagnostic and therapeutic patient information. Although many patients received complementary examinations and/or surgical interventions to verify the diagnosis or its therapy, it needs to be considered that cardiac MRI also contributed to the final diagnosis. Finally, the small sample size of only 37 patients with CHD limits statistical power. Therefore, replication in larger samples is needed in the future.

In conclusion, free-breathing non-contrast T1-TFE with compressed SENSE is robust at 3T and provides high diagnostic quality for assessment of the cardiothoracic vasculature in sedated young children with CHD. The ease of use without contrast injection and compensation for respiratory motion could facilitate widespread use in routine clinical practice. Further systematic investigations in larger cohorts are warranted to evaluate accuracy in various pathologies in comparison to clinical standard of care.

## Supporting information

S1 File S1) Baseline characteristics and performed diagnostic procedures considered for the reference standard; S2) Cross tables for sensitivity calculations of intracardiac structures; S3) Cross tables for sensitivity calculations of great extracardiac vessels; S4) Cross tables for sensitivity calculations of small extracardiac vessels; S5) Examples for the CMR image quality evaluation (Likert 1 to 5). S6) Estimates of regression coefficients with 95% confidence intervals and p-values of mixed models to compare contrast and slopes between the three MRI sequences; S7) Exemplary semi-automatic assessment of the signal intensity curves of the vessel wall and the lumen of the ascending aorta; S8) Estimated marginal means of the quantitative vessel assessment with two-sided 95% confidence intervals regarding A) contrast and B) vessel sharpness (slopes); S9) Vessel diameter measurement agreement between the two readers.
(DOCX)

S2 File De-identified data set. (XLSX)

S3 File Supporting movie showing time-resolved FB 2D CINE CS T1-TFE cardiovascular MRI of an exemplary patient (male, 3 years old) with a superior sinus venosus atrial septal defect. (MP4)
