# Supplementary material for: Non-contrast free-breathing 2D CINE compressed SENSE T1-TFE cardiovascular MRI at 3T in sedated young children for assessment of congenital heart disease
Source: PLoS One. 2024 Feb 8;19(2):e0297314. doi: 10.1371/journal.pone.0297314 (PMC10852305; doi:10.1371/journal.pone.0297314)
Supplement: S1 File — S1) Baseline characteristics and performed diagnostic procedures considered for the reference standard; S2) Cross tables for sensitivity calculations of intracardiac structures; S3) Cross tables for sensitivity calculations of great extracardiac vessels; S4) Cross tables for sensitivity calculations of small extracardiac vessels; S5) Examples for the CMR image quality evaluation (Likert 1 to 5). S6) Estimates of regression coefficients with 95% confidence intervals and p-values of mixed models to compare contrast and slopes between the three MRI sequences; S7) Exemplary semi-automatic assessment of the signal intensity curves of the vessel wall and the lumen of the ascending aorta; S8) Estimated marginal means of the quantitative vessel assessment with two-sided 95% confidence intervals regarding A) contrast and B) vessel sharpness (slopes); S9) Vessel diameter measurement agreement between the two readers. (DOCX) [file pone.0297314.s001.docx]

**Supplement**

***Supplementary Table S1)*** *Baseline characteristics and performed diagnostic procedures considered for the reference standard*

| Pat. | Sex | Age  [months] | Weight [kg] | Height [cm] | Mean heart rate [bpm]* | MRI scan duration  [h:min:s] | Main diagnosis | Surgery | Echo-cardiogram | Cardiac catheteri-zation | CT |
| --- | --- | --- | --- | --- | --- | --- | --- | --- | --- | --- | --- |
| #1 | m | 3 | 5.8 | 65 | 134 | 0:46:35 | ASD, VSD, hypoplastic aortic arch | x | x | x | - |
| #2 | f | 11 | 9.2 | 69 | 119 | 0:46:38 | ASD (upper sinus venosus type) with PAPVC into SVC | x | x | - |  |
| #3 | m | 5 | 7.5 | 68 | 78 | 0:42:53 | Heterotaxy Syndrome with left atrial isomerism and dextrocardia | x | x | x | x |
| #4 | m | 6 | 5.0 | 60 | 133 | 0:57:29 | cAVSD, DORV, Azygos continuation | x | x | x | - |
| #5 | f | 1 | 4.3 | 57 | 126 | 0:55:53 | Heterotaxy Syndrome with left atrial isomerism, Right-sided aortic arch, AVSD, Azygos continuation, PLSVC | x | x | x | - |
| #6 | f | 3 | 5.5 | 61 | 123 | 0:29:48 | ASD (upper sinus venosus type), PAPVC into SVC |  | x | - | - |
| #7 | m | 10 | 7.5 | 72 | 103 | 0:41:38 | S/P dTGA, RPA obstruction | x | x | x | - |
| #8 | m | 7 | 7.0 | 66 | 134 | 0:29:37 | LV-Dilation / DCM | - | x | x | - |
| #9 | m | 3 | 4.7 | 54 | 133 | 0:55:28 | S/P dTGA | x | x | x | - |
| #10 | m | 3 | 5.2 | 60 | 135 | 0:31:21 | cAVSD, PAPVC into the right atrium, hypoplastic aortic arch | x | x | x | - |
| #11 | m | 4 | 7.0 | 66 | 118 | 0:20:43 | VSD | x | x | - | - |
| #12 | f | 4 | 6.3 | 66 | 117 | 0:35:13 | VSD, pulmonary valve stenosis | - | x | - | - |
| #13 | m | 0 | 4.2 | 56 | 134 | 0:25:50 | DAA (right dominant) | x | x | - | - |
| #14 | f | 19 | 9.0 | 80 | 128 | 1:11:20 | ASD | x | x | - | - |
| #15 | m | 23 | 9.6 | 80 | 98 | 0:40:22 | MI, TI, left and right atrial enlargement,  pericardial effusion | - | x | x | - |
| #16 | f | 12 | 6.6 | 66 | 107 | 0:37:23 | ASD, VSD, left atrial isomerism, PLSVC, pulmonary artery aneurysm | x | x | x | - |
| #17 | m | 22 | 11.5 | 87 | 82 | 1:13:25 | ventricular tachycardia without structural heart defect | x | x | - | - |
| #18 | m | 14 | 8.7 | 74 | 98 | 0:37:09 | Alagille syndrome with bilateral peripheral pulmonary stenoses and MPA dilatation | - | x | - | - |
| #19 | f | 13 | 10.7 | 75 | 108 | 0:39:48 | ASD | - | x | - | - |
| #20 | f | 19 | 8.2 | 82 | 110 | 0:36:31 | ASD | x | x | - | - |
| #21 | f | 32 | 13.7 | 96 | 85 | 0:40:38 | S/P Ebstein's anomaly, pulmonary valve insufficiency with pulmonary artery aneurysm | x | x | x | - |
| #22 | m | 29 | 11.7 | 90 | 75 | 0:35:52 | AVSD, DORV, pulmonary atresia S/P Glenn Procedure | x | x | x | - |
| #23 | f | 29 | 15.4 | 97 | 88 | 0:25:46 | BAV | - | x | - | - |
| #24 | m | 33 | 14.0 | 100 | 83 | 0:46:19 | No structural heart defect, S/P patent ductus arteriosus | - | x | - | - |
| #25 | m | 29 | 11.8 | 94 | 91 | 0:39:25 | Scimitar Syndrome | x | x | - | - |
| #26 | f | 26 | 12.9 | 84 | 89 | 0:38:59 | Aortic valve stenosis, hypoplastic aortic arch | x | x | x | x |
| #27 | m | 47 | 17.3 | 105 | 72 | 0:51:55 | S/P dTGA, pulmonary valve stenosis | - | x | x | - |
| #28 | f | 37 | 10.0 | 87 | 88 | 0:34:17 | ASD, VSD, Unroofed Coronary Sinus, PLSVC | x | x | x | - |
| #29 | f | 47 | 15.8 | 101 | 103 | 0:25:26 | ASD, PAPVC into SVC | x | x | - | - |
| #30 | m | 37 | 15.0 | 97 | 75 | 0:50:36 | Dextrocardia, DORV, PLSVC | x | x | x | - |
| #31 | m | 40 | 16.4 | 104 | 76 | 0:57:33 | BAV, ISTA | x | x | x | - |
| #32 | m | 47 | 15.0 | 100 | 78 | 0:48:06 | ASD (upper sinus venosus type) with PAPVC into SVC | x | x | - | - |
| #33 | f | 44 | 12.0 | 92 | 98 | 0:28:00 | No structural heart defect, S/P ASD closure | x | x | - | - |
| #34 | f | 54 | 16.4 | 109 | 76 | 0:44:47 | Ebstein's anomaly | - | x | x | - |
| #35 | m | 48 | 20.0 | 113 | 95 | 0:59:05 | ASD (upper sinus venosus type) with PAPVC into RA | x | x | - | - |
| #36 | f | 53 | 13.0 | 100 | 78 | 0:48:57 | Heterotaxy Syndrome S/P cAVSD, S/P ISTA, PLSVC | x | x | x | - |
| #37 | f | 48 | 23.9 | 112 | 91 | 0:57:17 | S/P TOF, Right-sided aortic arch | x | x | x | - |

*Abbreviations: ASD: Atrial Septal Defect, BAV: Bicuspid Aortic Valve, (c)AVSD: (complete) Atrio-Ventricular Septal Defect, DAA: Double Aortic Arch, DORV: Double Outlet Right Ventricle, dTGA: d-Transposition of the Great Arteries, ISTA: Aortic Isthmus Stenosis, PLSVC persistent left superior vena cava, LPA: Left Pulmonary Artery, MI: Mitral Insufficiency, PAPVC: Partial Anomalous Pulmonary Venous Connection, RPA: Right Pulmonary Artery, S/P: Status Post, SVC: Superior Vena Cava, TI: Tricuspid Insufficiency, TOF: Tetralogy of Fallot, VSD: Ventricular Septal Defect. x = procedure was performed, - = procedure was not performed, * during MRI scan.*

***Supplementary Table S2)*** *Cross tables for sensitivity calculations of intracardiac structures*

*
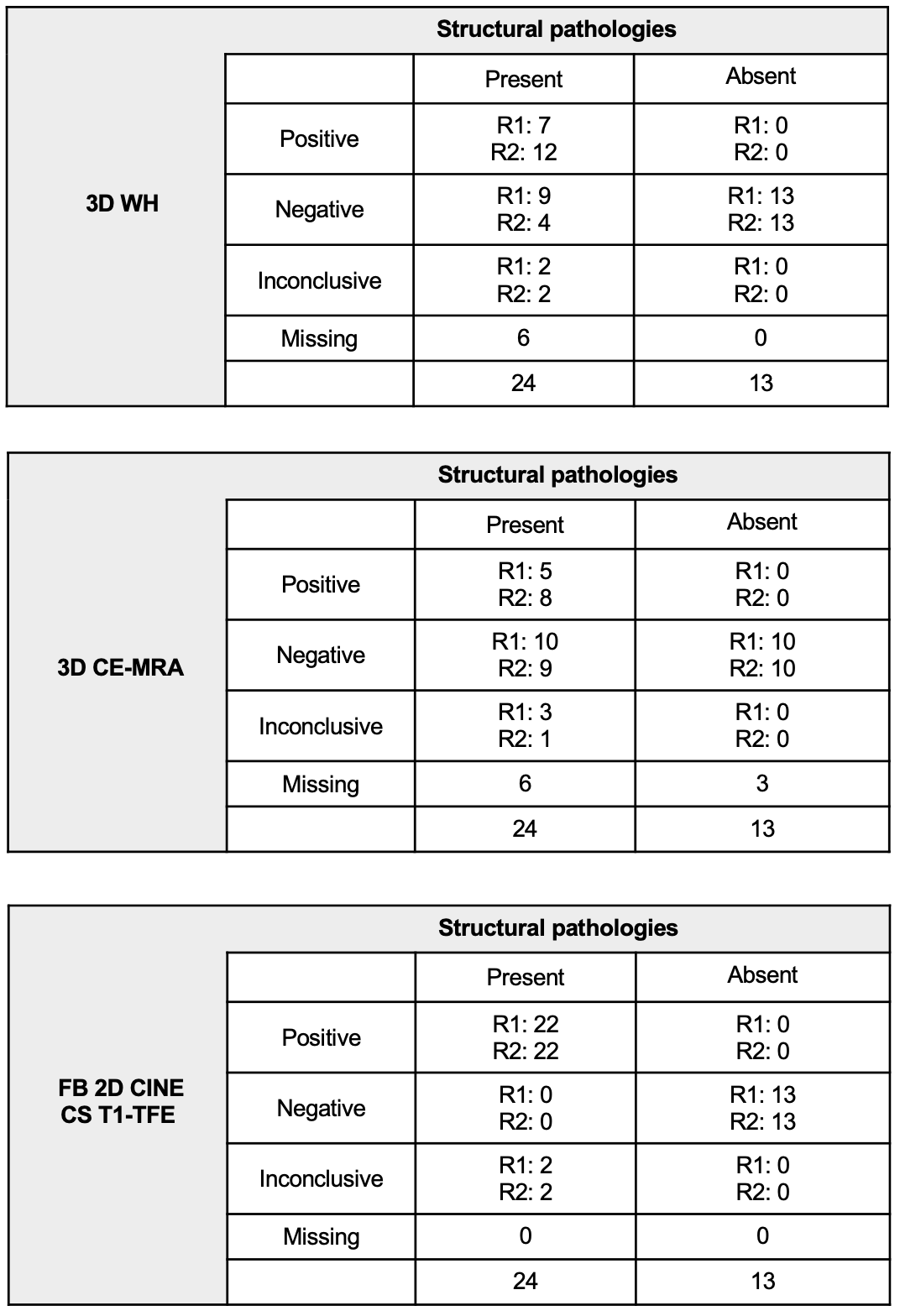
*

***Supplementary Table S3)*** *Cross tables for sensitivity calculations of great extracardiac vessels*

*
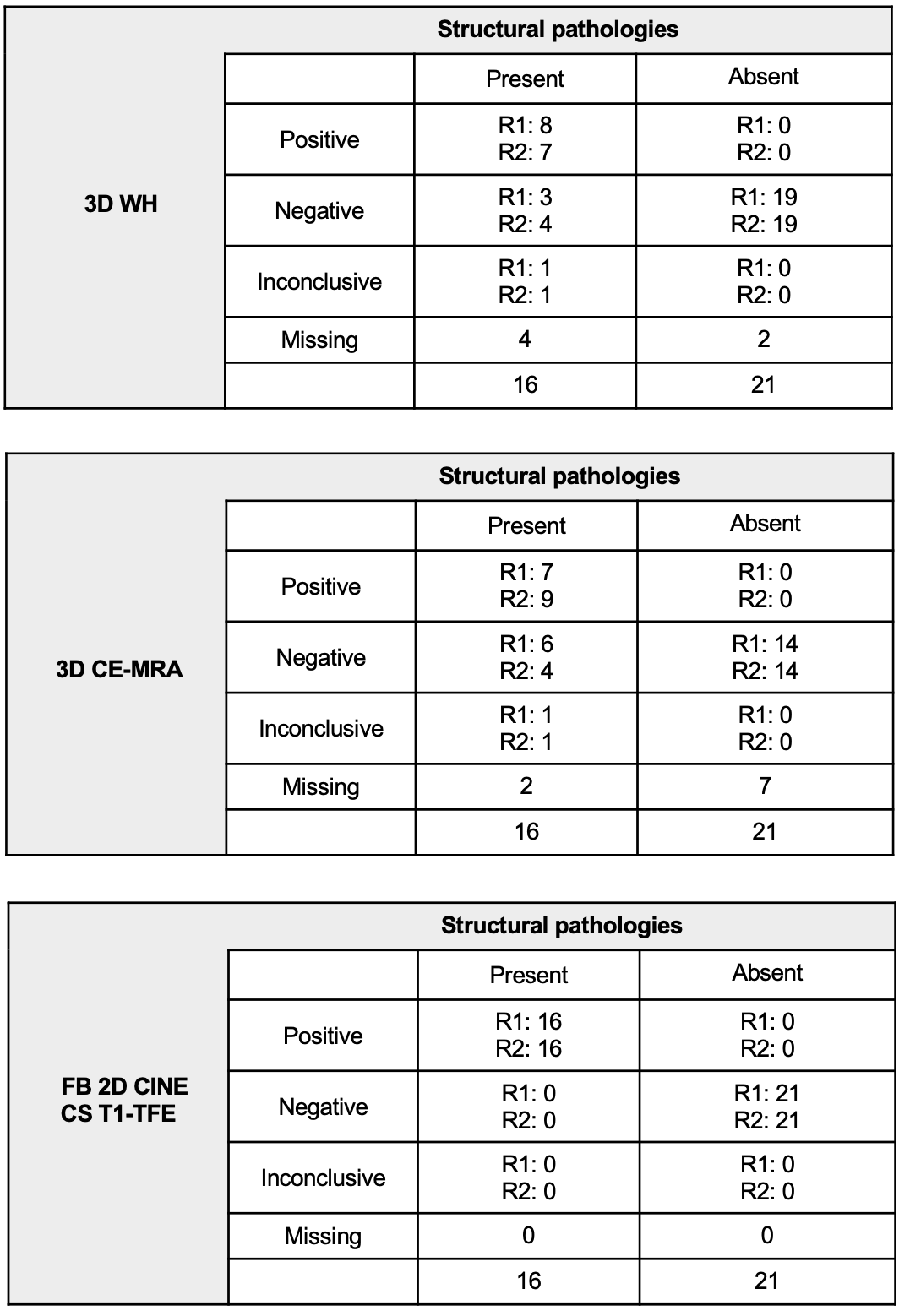
*

***Supplementary Table S4)*** *Cross tables for sensitivity calculations of small extracardiac vessels*

*
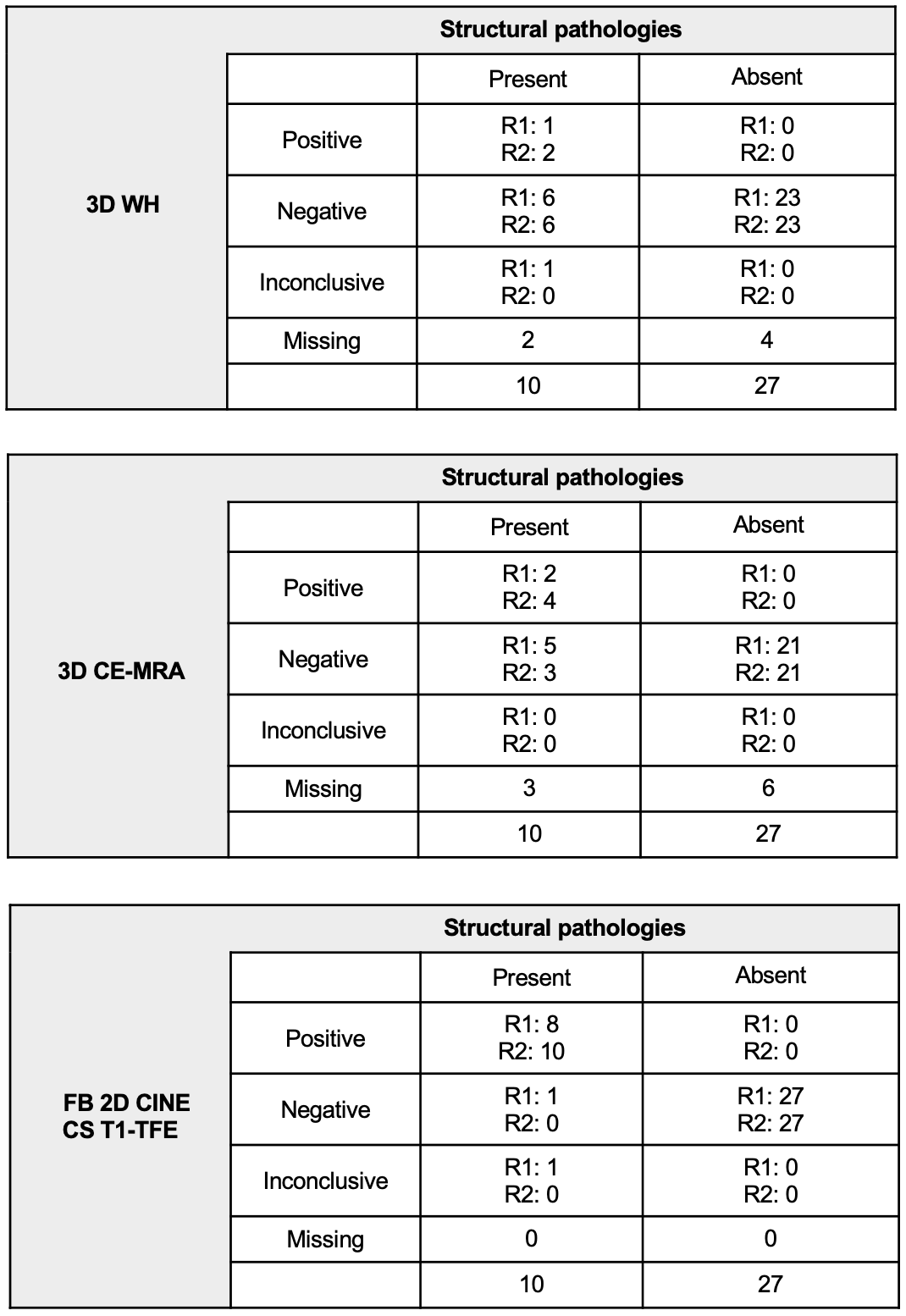
*

***Supplementary Table S5)*** *Examples for the CMR image quality evaluation (Likert 1 to 5)*

******

***Supplementary Table S6)*** *Estimates of regression coefficients with 95% confidence intervals and p-values of mixed models to compare contrast and slopes between the three MRI sequences. A random-intercept for each patient is included. MRI sequence, vessel and reader are included as fixed effects. Regarding the model with slope as dependent variable, side was additionally included as fixed effect. Unexplained residual variance within patients is denoted by* $\sigma^{2}$*, variance between patients is denoted by* $\tau_{00}$ *and number of patients is given with N.*

|  | Contrast | | | | | | Up- and down-slope | | | | | | | | | |  |  |
| --- | --- | --- | --- | --- | --- | --- | --- | --- | --- | --- | --- | --- | --- | --- | --- | --- | --- | --- |
| *Predictors* | *Estimates* | | | *CI* | | *p-value* | | *Estimates* | | | | | *CI* | | | *p-value* |  |  |
| Intercept | | 0.31 | | 0.27 – 0.36 | | < 0.001 | | | 312.32 | | | | 250.27 – 374.37 | | | < 0.001 |  |  |
| Sequence [3D CE-MRA] | | 0.14 | | 0.09 – 0.18 | | < 0.001 | | | | 107.32 | | | 64.25 – 150.40 | | | < 0.009 | |  |
| Sequence [FB 2D CINE CS T1-TFE] | | 0.36 | | 0.31 – 0.41 | | < 0.001 | | | | 501.58 | | | 458.51 – 544.65 | | | < 0.001 | |  |
| Vessel [Pulmonary trunc] | | 0.36 | | -0.08 – -0.00 | | < 0.001 | | | | -55.36 | | | -90.45 – -20.27 | | | 0.002 | |  |
| Reader [R2] | | -0.04 | | -0.05 – 0.02 | | 0.412 | | | | -19.18 | | | -54.24 – 15.89 | | | 0.283 | |  |
| Side  [right] | | |  | |  |  | | | | 8.92 | | | -26.15 – 43.98 | | | 0.618 | |  |
| Random effects | | |  | |  |  |  | | | | |  | | |  | | | |
| σ^2^ | | | 0.02 | |  |  | | | | | 41731.28 | | |  |  | | | |
| τ_00_ | | | 0.00 | |  |  | | | | | 11423.06 |  | | |  | | | |
| N | | | 22 | |  |  | | | | | 22 |  | | |  | | | |
| Observations | | | 262 | |  |  | | | | | 524 |  | | |  | | | |

***Supplementary Figure S7)*** *Exemplary semi-automatic assessment of the signal intensity curves of the vessel wall and the lumen of the ascending aorta in a 3-year-old boy. Compared to 3D WH-mDIXON (****A****) and 3D CE-MRA (****B****), FB 2D CINE CS T1-TFE (****C****) was characterized by a steeper slope of the vessel wall contours. a.u.= arbitrary unit.*

*
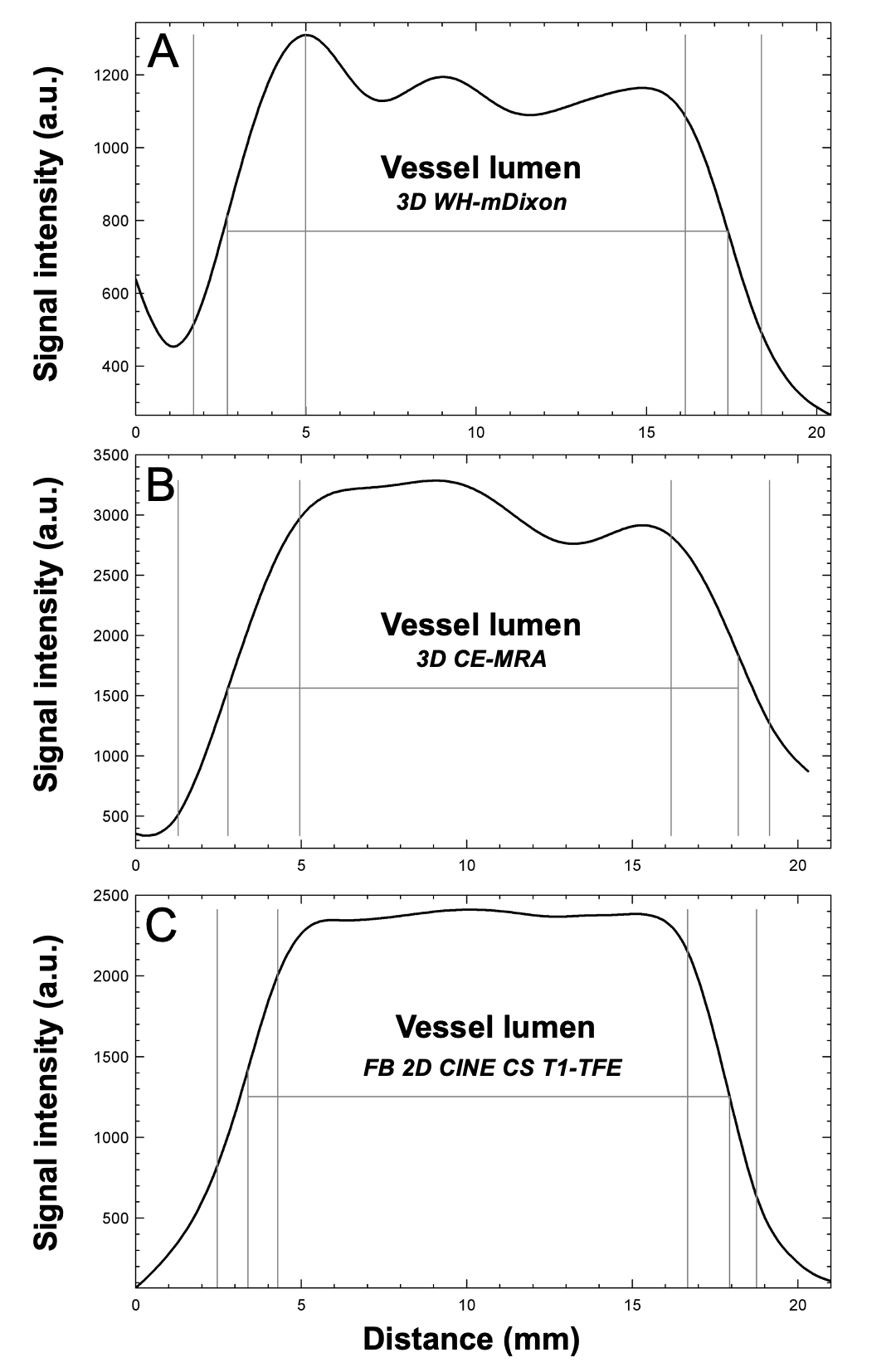
*

***Supplementary Figure S8)*** *Estimated marginal means of the quantitative vessel assessment with two-sided 95% confidence intervals regarding*

***A)*** *contrast and* ***B)*** *vessel sharpness (slopes)*

***
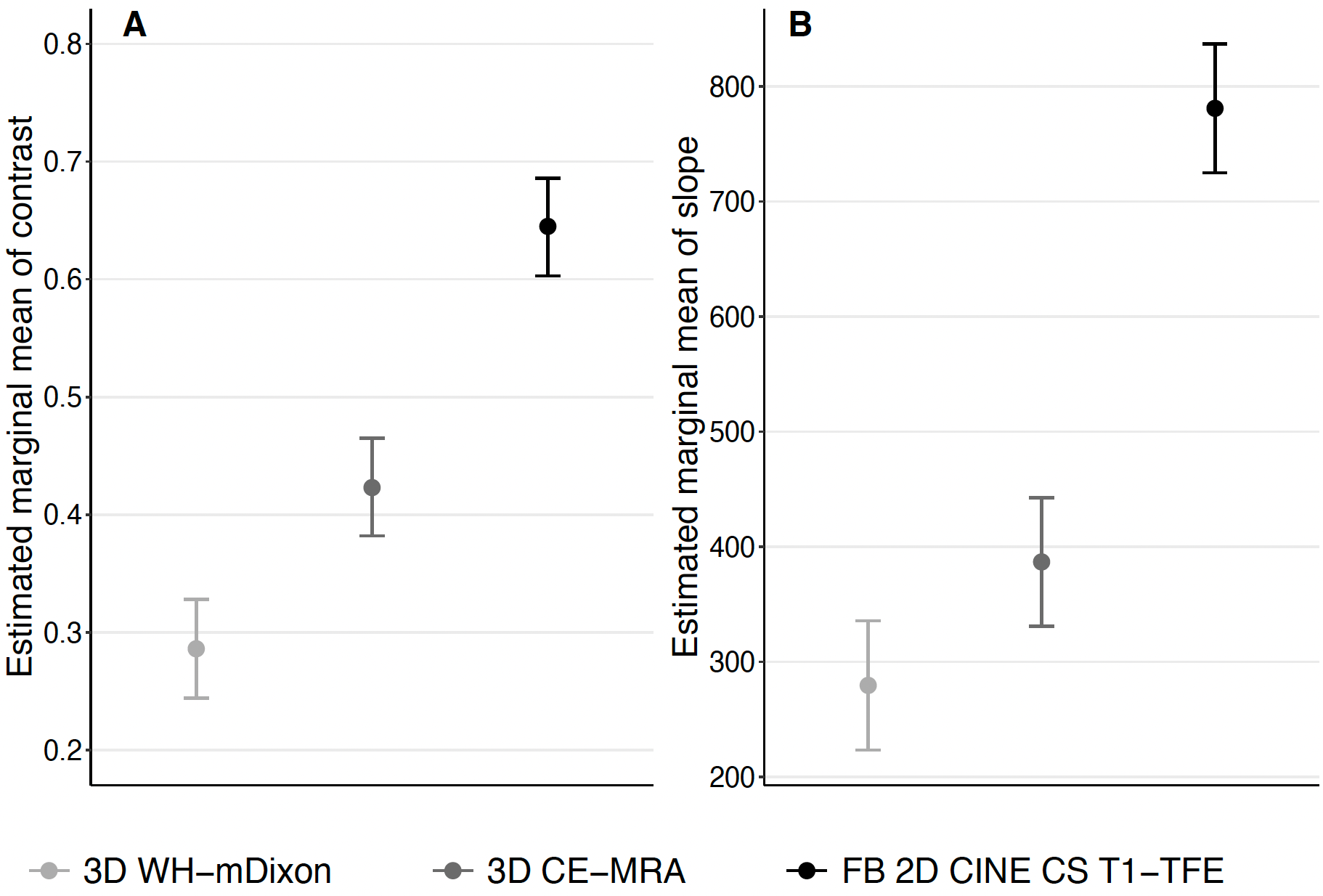
***

***Supplementary Table 9)*** *Vessel diameter measurement agreement between the two readers*

| Vascular structure | 3D WH  (N= 31) | | | 3D CE-MRA  (N= 28) | | FB 2D CINE CS T1-TFE  (N= 37) | |
| --- | --- | --- | --- | --- | --- | --- | --- |
|  | *Diameter*  *M ± SD [mm]* | *Krippendorff's alpha* | | *Diameter*  *M ± SD [mm]* | *Krippendorff's alpha* | *Diameter*  *M ± SD [mm]* | *Krippendorff's alpha* |
| 1. Sinuses of Valsalva | R1: 15.4 ± 3.7  R2: 15.6 ± 3.7 | | 0.94 (0.86 - 0.98) | R1: 15.2 ± 3.7  R2: 15.1 ± 3.6 | 0.93 (0.88 - 0.96) | R1: 15.1 ± 3.7  R2: 14.9 ± 3.6 | 0.96 (0.91 - 0.98) |
| 2. Sinotubular junction | R1: 11.5 ± 3.5  R2: 11.8 ± 4.0 | | 0.90 (0.77 - 0.96) | R1: 11.4 ± 2.9  R2: 11.3 ± 3.3 | 0.79 (0.63 - 0.90) | R1: 11.4 ± 3.4  R2: 11.3 ± 3.5 | 0.93 (0.86 - 0.97) |
| 3. Ascending aorta^1^ | R1: 12.4 ± 4.1  R2: 12.5 ± 4.4 | | 0.95 (0.86 - 0.98) | R1: 11.0 ± 3.1  R2: 11.1 ± 3.2 | 0.90 (0.78 - 0.96) | R1: 11.7 ± 4.0  R2: 11.7 ± 4.0 | 0.95 (0.90 - 0.98) |
| 4. Transverse aortic arc^2^ | R1: 8.9 ± 1.6  R2: 9.7 ± 2.9 | | 0.68 (0.37 - 0.87) | R1: 8.9 ± 1.9  R2: 9.0 ± 2.8 | 0.83 (0.73 - 0.90) | R1: 8.6 ± 2.7  R2: 8.4 ± 2.9 | 0.92 (0.84 - 0.96) |
| 5. Aortic isthmus | R1: 8.0 ± 1.6  R2: 8.6 ± 2.0 | | 0.84 (0.65 - 0.93) | R1: 7.7 ± 1.9  R2: 7.8 ± 2.0 | 0.78 (0.60 - 0.88) | R1: 7.6 ± 1.8  R2: 7.5 ± 1.9 | 0.80 (0.62 - 0.91) |
| 6. Distal arch | R1: 7.7 ± 1.7  R2: 7.6 ± 1.5 | | 0.75 (0.45 - 0.89) | R1: 7.8 ± 1.9  R2: 7.5 ± 1.7 | 0.76 (0.44 - 0.91) | R1: 7.6 ± 1.7  R2: 7.3 ± 1.6 | 0.82 (0.71 - 0.89) |
| 7. Descending aorta^1^ | R1: 7.2 ± 1.3  R2: 7.2 ± 1.5 | | 0.75 (0.54 - 0.87) | R1: 7.2 ± 1.6  R2: 7.0 ± 1.5 | 0.77 (0.52 - 0.89) | R1: 7.1 ± 1.4  R2: 6.8 ± 1.5 | 0.83 (0.71 - 0.89) |
| 8. Main pulmonary artery | R1: 14.5 ± 3.7  R2: 14.1 ± 3.6 | | 0.90 (0.76 - 0.95) | R1: 14.6 ± 3.3  R2: 13.8 ± 3.5 | 0.78 (0.48 - 0.91) | R1: 15.0 ± 5.0  R2: 14.9 ± 5.0 | 0.95 (0.91 - 0.98) |
| 9. Left pulmonary artery | R1: 8.7 ± 2.2  R2: 8.8 ± 2.3 | | 0.72 (0.42 - 0.88) | R1: 8.7 ± 1.8  R2: 9.1 ± 2.1 | 0.70 (0.46 - 0.86) | R1: 7.8 ± 2.3  R2: 7.8 ± 2.3 | 0.94 (0.88 - 0.97) |
| 10. Right pulmonary artery | R1: 8.4 ± 2.8  R2: 8.3 ± 2.5 | | 0.78 (0.46 - 0.89) | R1: 8.3 ± 2.5  R2: 8.8 ± 2.3 | 0.76 (0.35 - 0.92) | R1: 7.9 ± 2.6  R2: 7.8 ± 2.4 | 0.94 (0.86 - 0.97) |

*Krippendorff's alpha (0= no reliability, 1= perfect reliability) indicates agreement for diameters at ten predefined anatomic landmarks of the aorta (1.-7.) and pulmonary arteries (8.-10.). ^1^Diameter was assessed at the level of main pulmonary trunc; ^2^Diameter was assessed between brachiocephalic trunc and left common carotid artery. M: mean, SD: standard deviation, R1: reader 1, R2: reader 2.*
